# The Relationship between Hyperuricemia and Echocardiographic Parameters in Patients with Chronic Atrial Fibrillation

**DOI:** 10.3390/jcm12155034

**Published:** 2023-07-31

**Authors:** Mikel Jordhani, Majlinda Cafka, Joana Seiti, Vivencio Barrios

**Affiliations:** 1 Internal Medicine Department, Korça Regional Hospital, 7001 Korça, Albania; 2 Cardiovascular Diseases Department, UHC “Mother Teresa”, 1000 Tirana, Albania; lindareidea@yahoo.com (M.C.); joanaseiti@hotmail.com (J.S.); 3 Adult Cardiology Department, University Hospital Ramon y Cajal, 28034 Madrid, Spain; vivenciobarrios@gmail.com

**Keywords:** atrial fibrillation, uric acid, echocardiography, atrial dilation

## Abstract

*Purpose* Uric acid serves as a marker for cardiovascular risk and is often linked to inflammation and oxidative stress. There is evidence suggesting an association between uric acid and atrial fibrillation (AF), including its severity and occurrence of crises, as well as its involvement in cardiovascular mechanisms. The objective of this study was to assess the correlation between hyperuricemia and echocardiographic features in patients with chronic AF lasting for more than 5 years. *Methods* This case-control study involved 107 patients diagnosed with chronic non-valvular AF. Uric acid levels were measured in all patients, and they were divided into two groups: the first group consisted of 66 patients with hyperuricemia (>7.2 mg/dL), while the second group included 41 patients with normal uric acid levels. Echocardiography (TTE) was performed to evaluate each patient. Various clinical and echocardiographic parameters, such as left ventricle telediastolic (LVTDD) and telesystolic (LVTSD) diameters, left atrial diameter (LAD), aortic diameter (AoD), ejection fraction (EF), and pulmonary artery pressure (PAP), were analyzed. *Results* Binary logistic regression analysis revealed a statistically significant relationship between uric acid levels (>7.2 mg/dL) and LAD. For each unit increase in LAD, the probability of having hyperuricemia increased by 9% [odds ratio (OD): 0.91, 95% confidence interval (CI): 0.84—0.99]. A significant relationship was found between uric acid levels (>8 mg/dL) and LVESD (*p* = 0.045) as well as PAP (*p* = 0.006). For every unit increase in LVESD, the likelihood of having uric acid levels greater than 8 mg/dL increased by 22% [OD: 0.82, 95% CI: 0.67—0.99, b = −0.2]. Likewise, for each unit increase in PAP, the probability of having uric acid levels greater than 8 mg/dL was 9.4% [OD: 0.91, 95% CI: 0.86—0.97, b = −0.09]. *Conclusions* This study demonstrates that hyperuricemia has a significant relationship with the development of atrial remodeling, with an important association observed between hyperuricemia and an increase in left atrial diameter. Hyperuricemia is also correlated with an enlargement of the left ventricle end-systolic diameter and pulmonary artery pressure, showing a possible influence that hyperuricemia might have also left ventricle morphology and right ventricle function.

## 1. Introduction

Atrial fibrillation (AF) is the prevailing chronic cardiac arrhythmia encountered in clinical practice and represents the most common cause of hospitalization due to arrhythmias. Notably, approximately one-third of all arrhythmia-related hospitalizations are attributable to atrial fibrillation [1,2]. Current data reveal that the global burden of AF is estimated at 59.7 million, but its prevalence is expected to be more than double in the next 30 years, because of longer life expectancy and improved diagnosis [3,4]. Atrial fibrillation is associated with a five-fold risk for stroke, and it contributes significantly to cardiac morbidity and mortality [5,6].

Although not everything is known about the etiology of AF, it may involve diverse mechanisms, which can be categorized as either trigger-related or sustaining factors. Clinical phenotypes of persistent and permanent or paroxysmal AF exhibit distinct electrophysiological features and are influenced by various factors that affect the underlying substrate. These factors include valvular heart disease, heart failure, atrial dilatation, ischemia, sympathetic and vagal influences, inflammation, and fibrosis [7].

Recently, more focus on inflammation and oxidative stress has been given to the pathogenesis of AF [8]. Hyperuricemia represents one of the factors associated with oxidative stress and inflammation. Uric acid (UA) is the final product of purine catabolism, synthesized from hypoxanthine through the action of the enzyme xanthine oxidase and eliminated via renal excretion. Normal uric acid levels in healthy individuals range from 2.5–7.2 mg/dL. Hyperuricemia (HU) occurs when there is an increase in uric acid production or there is an impaired renal elimination [9,10]. Its role in cardiovascular disease and cardio-metabolic disorders is gaining importance, and hyperuricemia is associated with a higher risk of hypertension, coronary heart disease, and diabetes mellitus according to various studies [11,12].

According to the large Swedish cohort AMORIS, hyperuricemia is associated with an increased risk of AF, not only among people with cardiovascular disease and cardiovascular risk factors, but also among those without any preexisting cardiovascular disease [13].

Hyperuricemia, through the activation of the uric acid transporter (URAT1), has been hypothesized as a potential risk factor for atrial fibrillation and implicated in electrical remodeling. URAT1 is thought to play a significant role in reno-cardiovascular diseases associated with hyperuricemia [14]. Thus, it can be hypothesized that hyperuricemia inhibits the expression of ionic channels in atrial myocytes, thereby inducing electrical remodeling and contributing to the development of atrial fibrillation.

The aim of this study was to evaluate the relationship between hyperuricemia and echocardiographic parameters in patients with chronic atrial fibrillation (AF) lasting for 5 or more years, investigating their specific echocardiographic characteristics. By identifying and highlighting the echocardiographic features in these patients, this study will provide valuable insights for future clinical and treatment decisions.

## 2. Material and Methods

### 2.1. Study Population

This retrospective case-control study was conducted at the Cardiology Service of the “Mother Teresa” University Hospital Centre in Tirana, Albania. Clinical history data and echocardiographic parameters were collected from patients treated at our hospital. The study period encompassed the time from 1 December 2017 to 1 February 2019. All patients presenting with chronic atrial fibrillation (AF) were considered for inclusion, and only those who met the predefined inclusion criteria were included in the study.

The inclusion criteria of this study were:

(a) Patients with confirmed chronic non-valvular atrial fibrillation.
(b) Previous diagnosis of chronic non-valvular atrial fibrillation at least 5 years before the start of the study.

The following patients have been excluded from this study:

(a) Patients suffering from paroxysmal atrial fibrillation.
(b) Patients suffering from atrial fibrillation secondary to valvular heart disease.
(c) Patients suffering from heart failure with ejection fraction reduced (LVEF < 55%).
(d) Patients with dilated cardiomyopathy.
(e) Patients diagnosed with gout.
(f) Patients with chronic renal failure.
(g) Patients with elevated liver transaminases.
(h) Patients with uncontrolled arterial hypertension.
(i) Patients with diabetes mellitus.

### 2.2. Human and Technical Resources

The variables examined in this study encompassed both laboratory and echocardiographic parameters. Specifically, these included uricemia levels, as well as measurements such as the end-systolic (LVESD) and end-diastolic (LVEDD) diameters of the left ventricle, interventricular septal (IVS) thickness, left atrial diameter (LAD), shortening fraction (FS), left ventricle ejection fraction (EF), and pulmonary artery systolic pressure (PAsP).

Transthoracic echocardiography using a Philips HDI 5000 SonoCT machine with a 3.5 MHz probe was performed for all patients, encompassing the necessary windows and echocardiographic planes. The data collected were presented as variables along with their corresponding values.

Data extraction involved reviewing clinical records and echocardiographic reports for each patient, with all information recorded in individual echocardiographic reports. Subsequently, the collected data were organized into standardized variables and compiled into tables. Specific tables were created for each condition, with patient order determined by descending uric acid levels. Normal UA level were considered levels <7.2 mg/dL. Moderate hyperuricemia was considered when UA levels were 7.3–7.9 mg/dL, and severe hyperuricemia was considered when UA levels were ≥8 mg/dL.

The data collection table was divided into two groups: the control group (patients with normal uric acid levels) and the case group (patients with hyperuricemia). A backward stepwise conditional multivariate binary regression model was utilized for the comparison between the two groups, including only variables that showed statistical significance.

Additionally, patients were classified into two subgroups: those with normal uric acid levels or moderate hyperuricemia (combined as controls) and those with severe hyperuricemia (>8 mg/dL, classified as cases). The calculations were repeated using the backward stepwise conditional multivariate binary regression model. 
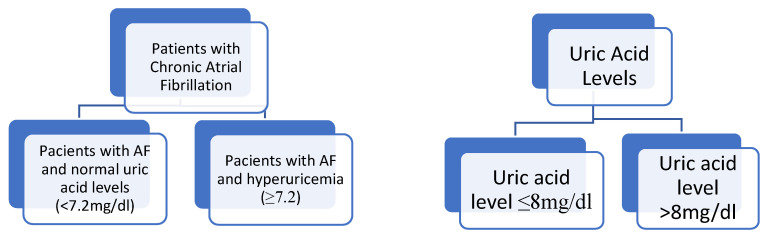


A *t*-test analysis was conducted to compare male and female patients. Statistical tests employed in this study included Student’s *t*-test, Mann–Whitney U test, Levene’s test, analysis of variance (ANOVA), and others. IBM SPSS Statistics 22 software was used for the statistical analysis. Statistical significance was defined as *p* < 0.05.

The authors declare that appropriate institutional review board approval was obtained or that the principles outlined in the Declaration of Helsinki were followed for all human or animal experimental investigations. In cases involving human subjects, informed consent was obtained from the participants involved.

## 3. Results

### 3.1. General Demographic and Clinical Characteristics

A total of 107 patients with chronic atrial fibrillation (AF) were enrolled in this study, comprising 48 (44.86%) women and 59 (55.14%) men. The mean age of the study population was 66.83 ± 7.07 years. Specifically, the average age for women was 66.1 ± 7.02 years, while for men, it was 67.4 ± 8.3 years. The average duration of chronic AF among the participants was 13.6 ± 5.12 years. The average disease duration for women was 14.2 ± 5.25 years, whereas for men, it was 13.05 ± 4.97 years. All these data are shown in Table 1.

### 3.2. Comparison of Groups Based on Uricemia Levels

The participants in this study were divided into two groups based on their uric acid levels, utilizing a cut-off point of 7.2 mg/dL as shown in Table 2. The first group (the control group) consisted of patients with chronic atrial fibrillation and normal uric acid levels (≤7.2 mg/dL), while the second group (case group) comprised patients with AF and hyperuricemia (>7.2 mg/dL).

Among the patients with chronic atrial fibrillation (AF) and normal uric acid levels, there were a total of 41 individuals, representing 38.3% of the entire sample. Within this subgroup, 22 were male (53.6%) and 19 were female (46.3%). The average age of these patients was 66.9 years, ranging from 50 to 83 years, with a standard deviation (SD) of 8.89. The mean duration of the disease in this group was 13.8 years, ranging from 5 to 25 years, with an SD of 5.33.

In terms of uric acid levels, the mean value for patients in this subgroup was 5.98 mg/dL, with a maximum value of 7.2 mg/dL and a minimum of 3.3 mg/dL (SD = 1.2). The mean left ventricular end-diastolic diameter (LVEDD) was 48.9 mm, ranging from 45 to 55 mm (SD = 1.7), while the mean left ventricular end-systolic diameter (LVESD) was 37.19 mm, with a range of 33 to 41 mm (SD = 2.14).

In terms of cardiac function, the average fractional shortening (FS) measured in these patients was 35%, ranging from 30% to 40% (SD = 3.1). The mean interventricular septal (IVS) thickness was 11 mm, ranging from 9 to 13 mm (SD = 1). The mean diameter of the left atrium (LAD) was 42.04 mm, with a range of 37 to 51 mm (SD = 2.85). The average diameter of the aorta (AoD) measured 37.63 mm, ranging from 35 to 41 mm (SD = 1.57). The mean left ventricle ejection fraction (LVEF) was 0.62, with a range of 0.56 to 0.7 (SD = 0.04). The average pulmonary artery systolic pressure (PAsP) was 22.68 mmHg, ranging from 10 to 37 mmHg (SD = 5.57).

Binary logistic regression analysis was conducted, with LAD and LVEF as independent variables, as shown in Table 3.

### 3.3. Group Analysis Based on Uricemia Cut-Off Point of 8 mg/dL

After the initial analysis, the sample was subsequently stratified based on uric acid levels, employing a cut-off value of 8 mg/dL. The control group comprised patients with normal or moderately elevated uric acid levels (<8 mg/dL), while the case group consisted of patients with significantly high uric acid levels (≥8 mg/dL). The findings obtained from this stratification are summarized and presented in Table 4.

**Figure 1:**
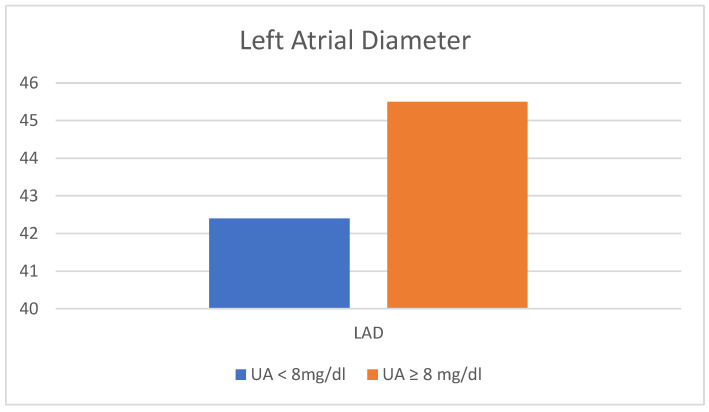
Comparison of the two groups in terms of left atrial diameter. LAD, left atrial diameter; UA, uric acid.

**Figure 2:**
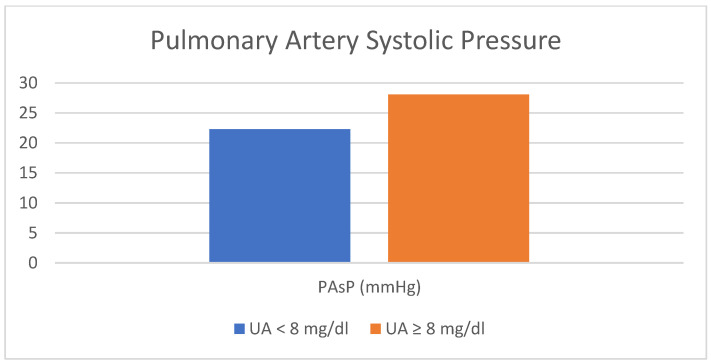
Comparison between two groups in terms of pulmonary artery systolic pressure. PAsP, pulmonary artery systolic pressure; UA, uric acid.

The comparison between the two groups in terms of LAD and PAsP is shown in Figure 1 and Figure 2 respectively.

Additionally, a binary logistic regression analysis was conducted using the uric acid level of 8 mg/dL as the designated cut-off point. This statistical analysis aimed to explore the relationship between various variables and the presence of uric acid levels exceeding this threshold. The results of this analysis are shown in Table 5 and Table 6.

## 4. Discussion

The primary objective of this study was to examine the correlation between hyperuricemia and left atrial diameter (LAD) in patients with chronic atrial fibrillation lasting for a minimum of 5 years. The findings of our study demonstrate a statistically significant relationship between uric acid levels and LAD in patients with chronic atrial fibrillation. Utilizing the backward stepwise conditional multivariate binary regression analysis, we observed a significant association between elevated uric acid levels (>7.2 mg/dL) and an increase in LAD. Specifically, for each unit increase in LAD, the likelihood of having uric acid levels exceeding 7.2 mg/dL increased by 9% (odds ratio: 0.91, 95% confidence interval: 0.84–0.99).

A study conducted by Chiu et al. investigating hyperuricemia in patients with chronic kidney disease found that hyperuricemia is linked to increased LAD and inappropriate left ventricular mass, including a reduced left ventricular ejection fraction (LVEF) in individuals with chronic kidney disease [15].

This observed correlation can be attributed to the elevated levels of oxidative stress and inflammation, which have also been implicated in the elevation of uric acid levels. However, it is important to exercise caution in interpreting the findings of this study due to the limitations imposed by the sample size.

Substantial evidence supports the hypothesis that atrial remodeling plays a central role in the development and perpetuation of chronic atrial fibrillation. Recent studies have shed light on the involvement of oxidative stress and inflammation in the pathophysiology of atrial fibrillation, although the causative relationship between these processes remains unclear. Inflammatory markers such as c-reactive protein (CRP) and interleukin-6 have been associated with an increased risk of atrial fibrillation and have been linked to chronic atrial fibrillation, left atrial dilation, and the promotion of a pro-thrombotic state [16,17].

Current epidemiological evidence indicates that uric acid functions as an autonomous risk factor for cardiovascular events and mortality among patients afflicted with conditions such as diabetes mellitus, arterial hypertension, heart failure, coronary disease, and post-cerebrovascular stroke recovery. Uric acid is a metabolic byproduct of purine metabolism and is produced by the enzyme xanthine oxidase (XO), which participates in diverse oxidative pathways [18]. It is recognized as a marker of oxidative stress and inflammation, with numerous studies already pointing to its involvement in cardiac remodeling.

Singh and Cleveland conducted a rigorous multivariate analysis to assess the risk of atrial fibrillation development in elderly patients diagnosed with gout. Their findings demonstrated a twofold increase in the risk of atrial fibrillation among individuals with gout [19]. Dudley et al. conducted an experimental study using an atrial tachy-pacemaker model, wherein they observed heightened xanthine oxidase (XO) activity in the left atrial appendage. This increased activity was effectively attenuated by the administration of oxypurinol, an XO inhibitor, resulting in a reduction in superoxide production [20]. Hyperuricemia, by activating the uric acid transporter 1 (URAT1), has been firmly established as an autonomous risk factor for atrial fibrillation and has been implicated in the process of electrical remodeling [21]. URAT1 is believed to have a substantial role in the pathogenesis of reno-cardiovascular diseases associated with hyperuricemia. It can be postulated that hyperuricemia exerts an inhibitory effect on the expression of ionic channels in atrial myocytes, thereby instigating electrical remodeling and contributing to the progression of atrial fibrillation.

Furthermore, multiple studies have provided evidence supporting the notion that uric acid acts as an independent precursor to the development of atrial fibrillation. Cohort studies have exhibited a significant increase in uric acid levels during the year preceding the diagnosis of atrial fibrillation, persisting into the first year following diagnosis. These findings imply the involvement of uric acid in the pathogenesis of atrial fibrillation [22]. In summary, it can be inferred that hyperuricemia induces atrial electrical remodeling through URAT1, thereby potentially initiating atrial fibrillation [22]. While atrial remodeling is recognized as a central factor in chronic atrial fibrillation, the precise role of uric acid in this process remains uncertain. Given the observational nature of our study, only associations with clinical implications can be proposed [23].

Furthermore, our study reveals a statistically significant correlation between blood uric acid levels and left ventricular ejection fraction (LVEF), suggesting that elevated uric acid levels are associated with lower LVEF (*p* = 0.046). This finding can be explained by the interrelated nature of left atrial and ventricular remodeling, wherein uric acid is believed to play a role. It is crucial to acknowledge the implications of uric acid in heart failure (HF), as several studies have demonstrated that hyperuricemia serves as an adverse prognostic factor in HF patients, with its effects potentially extending beyond atrial fibrillation. [21,23]. This finding is concordant even with the results of Chiu et al. as mentioned before [15].

Lastly, in this study, significant insights regarding the relationship between uric acid levels exceeding 8 mg/dL and two variables, specifically left ventricular end-systolic diameter (LVESD) (*p* = 0.045) and pulmonary artery systolic pressure (PAsP) (*p* = 0.006), have emerged through the utilization of binary logistic regression analysis with conditional backward steps.

The association observed between left ventricular end-systolic diameter and elevated uric acid levels can potentially be explained by the presumed remodeling of the left ventricle often associated with hyperuricemia. These findings align with the results of recent studies such as “Uric acid and the new onset of left ventricular hypertrophy: findings from the Pamela population” by Cuspidi et al., which demonstrated uric acid as a predictor of long-term echocardiographic changes, progressing from a normal left ventricular myocardial index to left ventricular hypertrophy, within a community sample [24].

The observed correlation between uric acid levels and pulmonary artery systolic pressure (PAsP) aligns with the findings of a meta-analysis conducted by Uk Kang et al. According to this meta-analysis, hyperuricemia has been identified as a risk factor for the subsequent development of pulmonary hypertension and is associated with an unfavorable prognosis [25]. As stated by Khosla et al., elevated levels of uric acid in individuals with pulmonary hypertension are believed to contribute to disease progression and prognosis. Existing evidence indicates that uric acid may impede acetylcholine-mediated vasodilation through its effects on the vascular endothelium [26]. Indeed, Zhang et al. conducted a study that revealed a significant association between uric acid (UA) levels and both the severity of idiopathic pulmonary hypertension (IPAH) and the extent of ventricular dysfunction [27]. This could potentially serve as a foundation for investigating the effects of uric acid on endothelial function.

According to Struthers et al. [28] and Kelkar et al. [29], uric acid lowering agents, especially allopurinol, might have beneficial effects on cardiac physiology, mainly by improving the key surrogates of endothelial dysfunction, vascular oxidative stress, myocardial ischemia, and left ventricular mass [28].

The primary objective of this observational study was to evaluate and analyze the relationship between uric acid levels and echocardiographic parameters in patients diagnosed with chronic atrial fibrillation. These findings suggest that uric acid levels may serve as a potential marker for assessing left atrial remodeling in this patient population.

Some of the limitations of this study are the sample size: a greater sample would give more consistent results, and patients on diuretics were not excluded (although diuretics may increase UA levels). Being an observational and retrospective study, it can assume only associations. It does not provide cause–consequence relationships.

Plasma uric acid level represents a low-cost, widely applicable, and easily accessible diagnostic test. If validated as a marker of atrial fibrillation severity, it could offer valuable diagnostic and prognostic information for patients with atrial fibrillation.

An intriguing avenue for future research would involve analyzing the impact of lowering uric acid levels on cardiac remodeling parameters in these patients. Conducting interventional studies to investigate the effects of uric acid reduction, utilizing agents such as allopurinol or febuxostat, would provide insights into the remodeling of the left atrium, the frequency of atrial fibrillation exacerbations, and the overall prognosis of the disease.

## 5. Conclusions

The present study provides evidence regarding the significant association between hyperuricemia and the development of atrial remodeling. Notably, a substantial correlation was observed between hyperuricemia and an augmentation in the left atrial diameter. Moreover, hyperuricemia exhibited a positive correlation with an enlargement of the left ventricular end-systolic diameter and pulmonary artery pressure, suggesting a potential influence of hyperuricemia on left ventricular morphology and right ventricular function. However, future studies on a larger cohort are needed to confirm these important findings.

## Figures and Tables

**Table 1:** General characteristics of the population.

Variables	Sex		*p* Value
	W, *n* = 48	M, *n* = 59	
Age	68.2 ± 7.0	65.7 ± 8.3	0.100
Disease duration	14.4 ± 5.3	13.1 ± 5.0	0.192
Uricemia	7.2 ± 1.3	7.3 ± 1.3	0.879
LVEDD	48.7 ± 3.7	49.9 ± 4.1	0.127
LVESD	37.1 ± 2.9	37.4 ± 2.4	0.565
FS	35.8 ± 3.0	34.3 ± 3.6	0.021
IVS	10.8 ± 1.0	10.9 ± 1.2	0.767
LAD	43.4 ± 4.9	43.1 ± 4.8	0.739
AoD	37.0 ± 1.8	37.0 ± 3.2	0.905
LVEF	0.6 ± 0.0	0.6 ± 0.0	0.340
PAsP	23.9 ± 7.8	23.9 ± 7.6	0.972

M, men; W, women; LVEDD, left ventricle end diastolic diameter; LVESD, left ventricle end systolic diameter; FS shortening fraction; IVS interventricular septum thickness; LAD, left atrial diameter; AoD, aortic diameter; LVEF, left ventricle ejection fraction; PAsP, pulmonary artery systolic pressure.

**Table 2:** Comparison of demographic and echocardiographic data between patients with normal uricemia and hyperuricemia.

Variables	Uric Acid Levels		Value *p*
	≥7.2 mg/dL, *n* = 66	<7.2 mg/dL, *n* = 41	
Age	66.7 ± 7.1	67.0 ± 8.9	0.881
Disease duration	13.5 ± 5.0	13.8 ± 5.3	0.789
Uricemia	8.10 ± 0.4	6.0 ± 1.2	<0.001
LVEDD	49.7 ± 4.8	48.9 ± 1.7	0.251
LVESD	37.3 ± 2.9	37.2 ± 2.1	0.802
FS	35.0 ± 3.6	35.0 ± 3.1	0.982
IVS	10.8 ± 1.2	11.0 ± 1.0	0.342
LAD	44.0 ± 5.6	42.0 ± 2.9	0.021
AoD	36.6 ± 3.1	37.6 ± 1.6	0.023
LVEF	0.6 ± 0.0	0.6 ± 0.0	0.071
PAsP	24.6 ± 8.7	22.7 ± 5.6	0.160
Sex (M)	37(56.1%)	22 (53.7%)	0.808

M, men; W, women; LVEDD, left ventricle end diastolic diameter; LVESD, left ventricle end systolic diameter; FS shortening fraction; IVS interventricular septum thickness; LAD, left atrial diameter; AoD, aortic diameter; LVEF, left ventricle ejection fraction; PAsP, systolic pulmonary artery pressure.

**Table 3:** Binary logistic regression in terms of ejection fraction and left atrial diameter.

							Variables Ein the Equation				
		B		S.E.		Wald	df	Sig.	Exp(B)	95% C.I. for EXP(B)	
										Lower	Upper
Step 1 ^a^	LVEF	6.787		5.080		1.785	1	0.182	886.048	0.042	1.869 × 10^7^
	LAD	−0.072		0.045		2.565	1	0.109	0.930	0.852	1.016
	Constant	−1.577		4.049		0.152	1	0.697	0.207		

^a^ Variable(s) entered on step 1: LVEF, LAD. LVEF, left ventricle ejection fraction, LAD, left atrial diameter.

**Table 4:** Comparison of demographic and echocardiographic data between patients with uricemia >8 mg/dL and <8 mg/dL.

Variables	Uricemia		Value *p*
	≥8 mg/dL, *n* = 30	<8 mg/dL, *n* = 77	
Age	67.8 ± 6.9 [61.8–72.3]	66.5 ± 8.1 [59.0–72.5]	0.441
Duration of the disease	15.0 ± 5.5 [11.0–20.0]	13.1 ± 4.9 [10.0–15.0]	0.085
Uricemia	8.4 ± 0.3 [8.1–8.6]	6.8 ± 1.3 [6.5–7.8]	<0.001
LVEDD	49.7 ± 5.3 [44.8–54.0]	49.3 ± 3.3 [48.0–51.0]	0.715
LVESD	38.4 ± 2.8 [36.0–40.3]	36.8 ± 2.4 [35.0–38.5]	0.004
FS	35.2 ± 4.6 [34.0–38.0]	34.9 ± 2.9 [34.0–37.0]	0.742
IVS	10.9 ± 1.2 [10.0–12.0]	10.9 ± 1.1 [10.0–12.0]	0.950
LAD	45.5 ± 5.0 [43.0–48.3]	42.4 ± 4.5 [40.0–45.5]	0.002
AoD	37.7 ± 2.9 [36.0–39.3]	36.7 ± 2.5 [35.0–38.0]	0.084
LVEF	0.6 ± 0.1 [0.58–0.64]	0.6 ± 0.03 [0.59–0.65]	0.046
PAsP	28.1 ± 8.9 [21.0–35.5]	22.3 ± 6.5 [19.0–25.5]	0.002
Sex (H)	16 (53.3%)	43 (55.8%)	0.815

M, men; W, women; LVEDD, left ventricular end-diastolic diameter; LVESD, left ventricular end systolic diameter; FS, shortening fraction; IVS, size of the interventricular septum; LAD, left atrial diameter; AoD, aortic diameter; LVEF, left ventricle ejection fraction; PAsP, pulmonary artery systolic pressure.

**Table 5:** Comparison between the two groups using the uric acid value of 8 mg/dL as the cut-off point.

	Observed		Predicted		
			Uricemia 8		Percentage Correct
			≥8 mg/dL	<8 mg/dL	
Step 1	Uricemia 8	≥ 8 mg/dL	8	22	26.7
		< 8 mg/dL	3	74	96.1
	Overall Percentage				76.6
Step 2	Uricemia 8	≥ 8 mg/dL	10	20	33.3
		< 8 mg/dL	5	72	93.5
	Overall Percentage				76.6

**Table 6:** Conditional stepwise binary logistic regression.

		B	S.E.	Wald	df	Sig.	Exp(B)	95% C.I. for EXP(B)	
								Lower	Upper
Step 1 ^a^	PAsP	−0.104	0.032	10.777	1	0.001	0.901	0.847	0.959
	Constant	3.543	0.848	17.455	1	0.000	34.581		
Step 2 ^b^	LVESD	−0.200	0.100	4.001	1	0.045	0.819	0.673	0.996
	PAsP	−0.090	0.033	7.580	1	0.006	0.914	0.857	0.974
	Constant	10.699	3.765	8.074	1	0.004	44328.809		

^a^ Variable(s) entered on step 1: PAsP. ^b^ Variable(s) entered on step 2: TS_VM. PAsP, pulmonary artery systolic pressure; LVESD, end systolic diameter of the left ventricle.

## Data Availability

The data that support the findings of this study are available from the corresponding author, M.J., upon reasonable request.
